# Supplementary material for: Cell-Based Models of ‘Cytokine Release Syndrome’ Endorse CD40L and Granulocyte–Macrophage Colony-Stimulating Factor Knockout in Chimeric Antigen Receptor T Cells as Mitigation Strategy
Source: Cells. 2023 Nov 6;12(21):2581. doi: 10.3390/cells12212581 (PMC10649043; doi:10.3390/cells12212581)
Supplement: Supplementary file 1 [file cells-12-02581-s001.zip › Supplementary_Figures.pdf]

Supplementary Materials

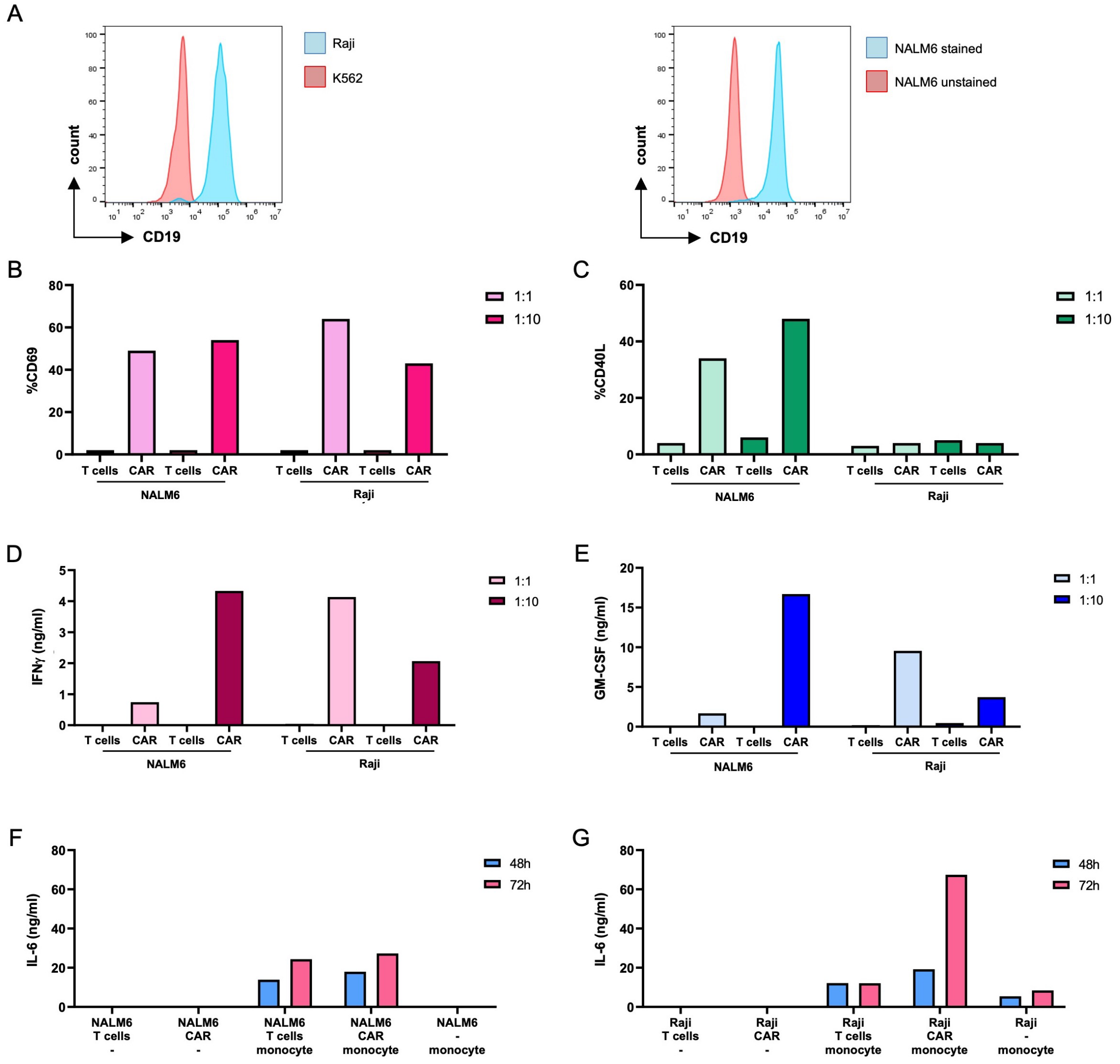

Figure S1. Identification of optimal effector-to-target cell ratio. (**A**) CD19 expression levels. Flow cytometric analysis of CD19 expression of K562, Raji, and NALM6 cells. **(B-C)** Cellular markers. CD19-targeted CAR T cells were seeded with $CD19^+$ NALM6 or $CD19^+$ Raji target cells either at 1:1 or 1:10 effector-to-target (E:T) cell ratios. The fraction of CD69- or CD40L-positive CAR T cells was evaluated 4 h later in the population of $\Delta LNGFR^+$ cells. **(D-E)** Secreted markers. Supernatants were collected after 24 h and concentrations of IFN-γ and GM-CSF determined by cytometric bead array (CBA). Non-transduced T cells served as negative controls. (**F-G**) IL-6 release. CD19-targeted CAR T cells were co-cultured with monocytes from the same donor and CD19+ target cells, either NALM6 cells at a 1:1:10 ratio (F), or Raji cells at 1:1:1 ratio (G). Supernatants were collected 48h and 72h later and concentrations of IL-6 determined by CBA.

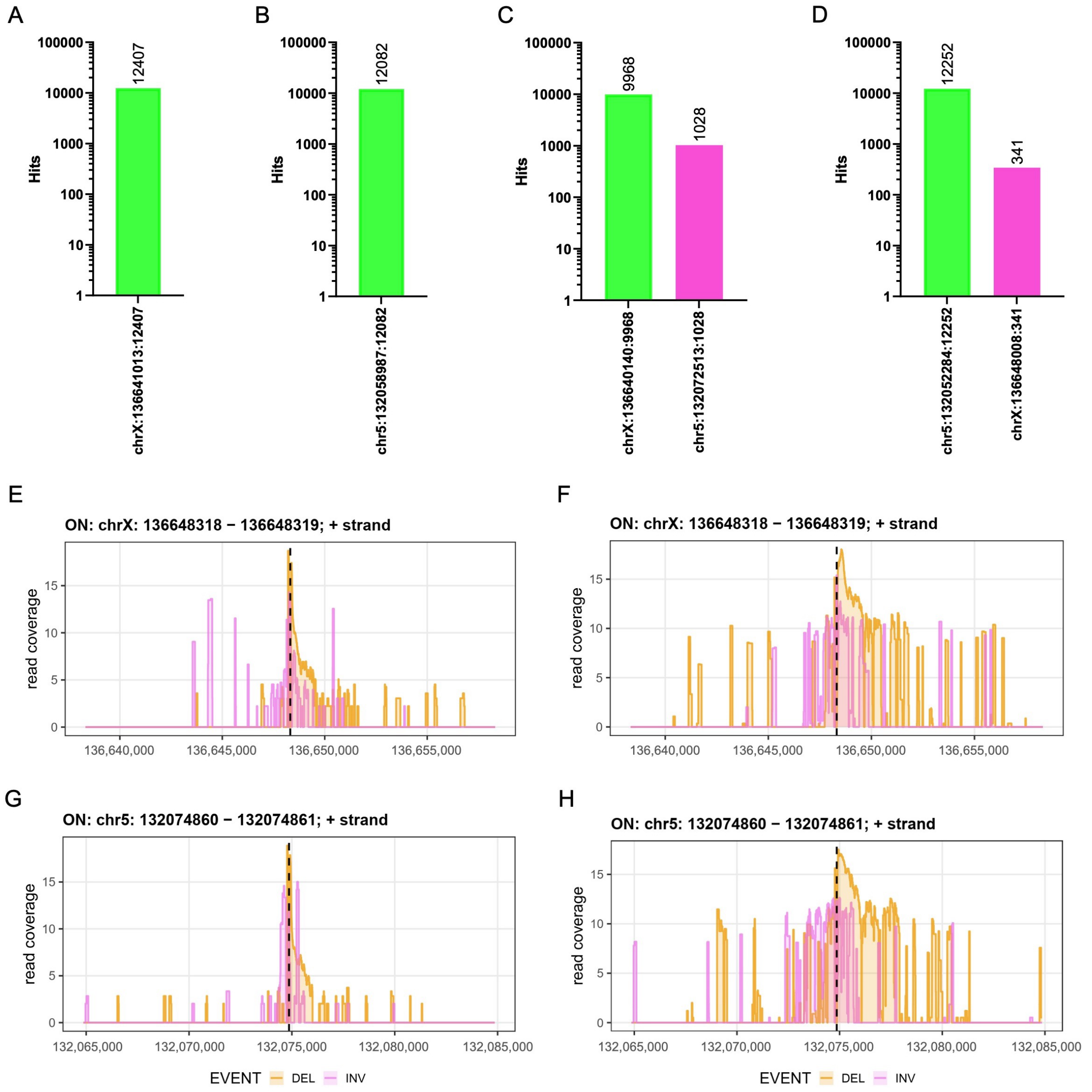

Figure S2. Evaluation of off-target effects. (A-D) CAST-Seq hits. Relative quantification of CAST-Seq hits. The plots indicate the number of hits for each chromosomal rearrangement in CAR T cells that were edited individually at either the *CD40L* (A) or *CSF2* locus (B), or simultaneously using either *CD40L* (C) or *CSF2 (*D) as an anchor for CAST-Seq. Aberrations at the on-target site are indicated in green, off-target mediated translocations in red (absent), and translocations between the *CD40L* and *CSF2* target sites in purple. **(E-H)** On-target aberrations. Coverage plots show CAST-Seq reads mapped to a +/- 10 kb region around the *CD40L* (E, untreated control; F, double edited cells) or *CSF2* (G, untreated control; H, double edited cells) target sites. Sequencing direction is from left to right. The x-axis indicates the chromosomal coordinates, the y-axis the log2 read count per million, and the dotted line the cleavage site. Deletions (DEL) are shown in orange, inversions (INV) in purple.

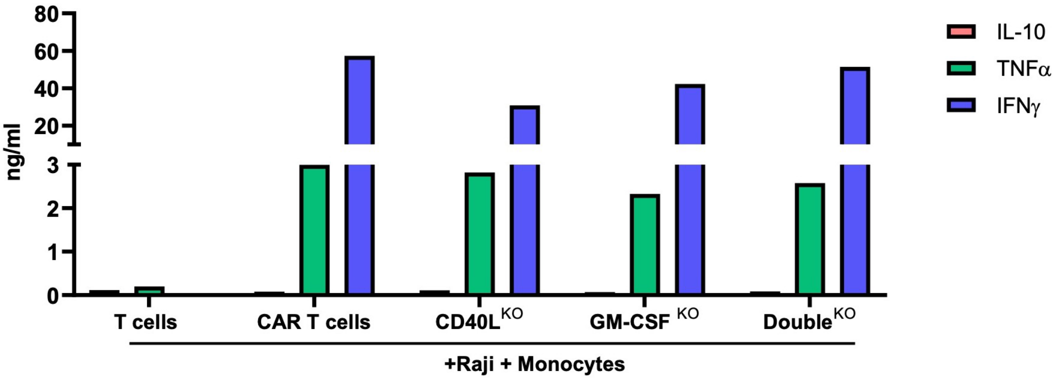

Figure S3. Cytokine release profile. Shown are the concentrations of IL-10, TNFα and IFNγ that we determined by cytometric bead array in the supernatants of monocytes that were co-cultured with edited or non-edited CD19-targeted CAR T cells and Raji cells at a 1:1:1 ratio. Non-transduced T cells served as negative controls.
